# Skeletal muscle optoacoustics reveals patterns of circulatory function and oxygen metabolism during exercise

**DOI:** 10.1016/j.pacs.2023.100468

**Published:** 2023-02-25

**Authors:** Angelos Karlas, Nikolina-Alexia Fasoula, Nikoletta Katsouli, Michael Kallmayer, Sabine Sieber, Sebastian Schmidt, Evangelos Liapis, Martin Halle, Hans-Henning Eckstein, Vasilis Ntziachristos

**Affiliations:** a Institute of Biological and Medical Imaging, Helmholtz Zentrum München, Neuherberg, Germany; b Chair of Biological Imaging at the Central Institute for Translational Cancer Research (TranslaTUM), School of Medicine, Technical University of Munich, Munich, Germany; c Department for Vascular and Endovascular Surgery, Klinikum rechts der Isar, Technical University of Munich, Munich, Germany; d DZHK (German Centre for Cardiovascular Research), partner site Munich Heart Alliance, Munich, Germany; e Department of Prevention and Sports Medicine, Klinikum rechts der Isar, Technical University of Munich, Munich, Germany

**Keywords:** MSOT, Multispectral Optoacoustic Tomography, HbO_2_, Oxygenated Hemoglobin, Hb, Deoxygenated Hemoglobin, TBV, Total Blood Volume, PAD, Peripheral Arterial Disease, MVC, Maximum Voluntary Contraction, Multispectral optoacoustic tomography, MSOT, Photoacoustics, Musculoskeletal physiology, Hemodynamics, Oxygenation, Metabolic imaging

## Abstract

Imaging skeletal muscle function and metabolism, as reported by local hemodynamics and oxygen kinetics, can elucidate muscle performance, severity of an underlying disease or outcome of a treatment. Herein, we used multispectral optoacoustic tomography (MSOT) to image hemodynamics and oxygen kinetics within muscle during exercise. Four healthy volunteers underwent three different hand-grip exercise challenges (60s isometric, 120s intermittent isometric and 60s isotonic). During isometric contraction, MSOT showed a decrease of HbO_2_, Hb and total blood volume (TBV), followed by a prominent increase after the end of contraction. Corresponding hemodynamic behaviors were recorded during the intermittent isometric and isotonic exercises. A more detailed analysis of MSOT readouts revealed insights into arteriovenous oxygen differences and muscle oxygen consumption during all exercise schemes. These results demonstrate an excellent capability of visualizing both circulatory function and oxygen metabolism within skeletal muscle under exercise, with great potential implications for muscle research, including relevant disease diagnostics.

## 1 Introduction

Muscle contraction, the hallmark of exercise, reflects the conversion of metabolic energy into mechanical function. Accurate non-invasive monitoring of such processes is crucial for the objective assessment not only of the muscle function itself but also of general body performance. Relevant long pending questions may well be answered by measuring muscle metabolism, as reflected in its perfusion and oxygenation changes during exercise [1], questions such as: i) are the main determinants of the maximal oxygen transport and uptake—both major metrics of exercise capacity—the central adaptations to exercise or the local muscle phenomena [2], [3] or, ii) what is the contribution of skeletal muscle in the definition of basic body energy expenditure, as a barometer of obesity risk?

Muscle measurements are also useful in everyday practice. For example, in health-related exercise, measuring normal muscle function and metabolism is essential for the evaluation of an athlete’s performance and corresponding adjustment of the training program [4]. In disease conditions, such as peripheral arterial disease (PAD), neuromuscular disorders, and diabetes, accurate monitoring of muscle function and metabolism may enable the quantification of exercise tolerance and the optimization of therapeutic schemes [5], [6]. Thus, muscle measurements of circulatory function (hemodynamics) and oxygen kinetics could give not only insights in local muscle physiology and pathology but also serve as a window to the mechanisms regulating the coupling between the systemic response to exercise and local muscle metabolism.

Several techniques have been used for non-invasive monitoring of muscle function and metabolism for diagnostic purposes, therapy evaluation, or physiology studies. Most of the commonly used methods (e.g., contrast-enhanced ultrasound [CEUS], positron emission tomography [PET], single photon emission computed tomography [SPECT], contrast-enhanced magnetic resonance imaging [MRI]) visualize muscle function and metabolism using exogenous tracers that are taken up by the muscle. For example, the intramuscular circulation of injected microbubbles was imaged using CEUS on muscle in patients with PAD and diabetes, showing disturbed muscle perfusion after exercise [7], [8]. While CEUS can provide useful insights into muscle function, it provides no information about muscle metabolism, and does not allow for observations during prolonged functional challenges (e.g., exercise tests) since the agent is cleared from the vasculature by the liver, immune system, or by ultrasound-induced mechanical destruction, in only three to five minutes [9]. Moreover, the need for injection and venous cannulation increases discomfort for the patient. Similarly, nuclear medicine techniques such as PET or SPECT can also be used to characterize muscle perfusion and function indirectly through the clearance dynamics of injected radioactive tracers [10], [11]. However, both PET and SPECT suffer from low image resolution (≈ 4 mm) and expose patients to ionizing radiation [12].

Non-invasive muscle monitoring can also be achieved by imaging intramuscular levels of endogenous chemical metabolites, which is possible with magnetic resonance spectroscopy (MRS), muscle functional magnetic resonance imaging (mfMRI), and chemical exchange saturation transfer-magnetic resonance imaging (CEST-MRI) [13], [14], [15]. Several of these techniques have been employed for assessing muscle function and metabolism, most of which are non-ionizing and do not necessitate the use of injected contrast agents. However, such methods are based on indirect readouts of metabolites, without direct monitoring of perfusion and oxygenation/aerobic contrast. Furthermore, these techniques require elaborate infrastructure and expensive equipment, may lead to patient inconvenience (due to long examination times or claustrophobia [16]), and are generally low-throughput. Therefore, such magnetic resonance imaging modalities are not well-suited for large-scale functional studies and disseminated use.

Finally, muscle function and metabolism can also be imaged by measuring perfusion and oxygen kinetics, using techniques such near-infrared spectroscopy (NIRS) and diffuse optical tomography (DOT) [14], [17], [18]. These optical methods take advantage of the changes in the optical properties of hemoglobin according to its oxygenation status. While they have shown great potential for imaging muscle perfusion and oxygen metabolism based directly on hemoglobin contrast, the limited accuracy and resolution (5–10 mm) due to light scattering has prevented their wide-spread use [19], [20].

Multispectral optoacoustic tomography (MSOT) is able to overcome the above such limitations as it has the potential to provide imaging of muscle function and metabolism with high spatial (≈ 200–300 µm) and temporal (≈ 25–50 Hz) resolution—without the need for contrast agents—and high portability due to the newly available hand-held configurations. More importantly, hand-held MSOT can achieve detailed and real-time imaging of intramuscular oxygen kinetics and circulation mechanics based only on hemoglobin contrast (oxy- [HbO_2_] and deoxygenated [Hb]) and fluctuations over time, providing direct access to ‘aerobic’ processes. Contrary to purely optical techniques, MSOT employs fast light pulses to produce and sense ultrasound waves and is therefore unaffected by light scattering. MSOT, especially after recent image quality improvements [21], can be employed at depths of up to 3–4 cm and has already been used in the study of tumors and cardiovascular, metabolic, endocrine, neuromuscular and inflammatory diseases [20], [22], [23], [24], [25], [26], [27], [28]. Particularly relevant for the current study, MSOT has shown great potential for imaging skeletal muscle under disturbed blood flow conditions (arterial and venous occlusion) [29], [30], as well as before and after cycling exercise [31].

In this study, we explore the promising capability of hand-held MSOT for direct and label-free imaging HbO_2_ and Hb in real-time to investigate muscle function and metabolism during different types of exercise. We use MSOT to discriminate between HbO_2_ and Hb and resolve their dynamics over time and provide invaluable insights into the mechanics of circulation and the metabolism of oxygen within muscle during isometric, intermittent isometric, and isotonic exercise. We show that MSOT provides high-resolution imaging of ‘aerobic’ processes in a non-invasive and label-free, manner reaching depths of 3–4 cm in muscle tissue. Application of this approach on healthy volunteers demonstrates great potential for research and diagnostics of muscle exercise physiology in health and disease and could become a new method of choice for exercise performance evaluation.

## 2 Materials and methods

### 2.1 Study design and subject preparation

All participants signed an informed consent before included in the study. The study was approved by the ethics committee of the medical faculty of the Technical University of Munich (Protocol No: 324/21S). All subjects (n = 4, 2 males, 2 females; mean age 35, range 34–36) were non-smokers, normotensive (blood pressure 110–120/75–80 mmHg) and of average weight and level of fitness. Subjects with any condition that may affect muscle function were not included. Also, the included participants were not taking any medication and were asked to avoid exercise for at least 8 h before the measurements to avoid any effect on the MSOT muscle measurements. Measurements took place in a quiet room with normal temperature (T ≈ 23 °C). Subjects were seated in a comfortable chair and the handgrip maximum voluntary contraction (MVC) of the dominant hand was measured using a hand-held digital dynamometer. MVC was measured three consecutive times (at 5 min intervals) and the average measured value was recorded as the subject’s handgrip MVC. Each subject was then provided with a handgrip workload in the dominant hand corresponding to 40 % of the MVC. The hand-held MSOT probe was placed over the dorsolateral region of the dominant forearm and the position was marked with permanent ink. The muscle was imaged over the same position during all exercise challenges.

### 2.2 Imaging setup and data acquisition

**Fig. 1:**
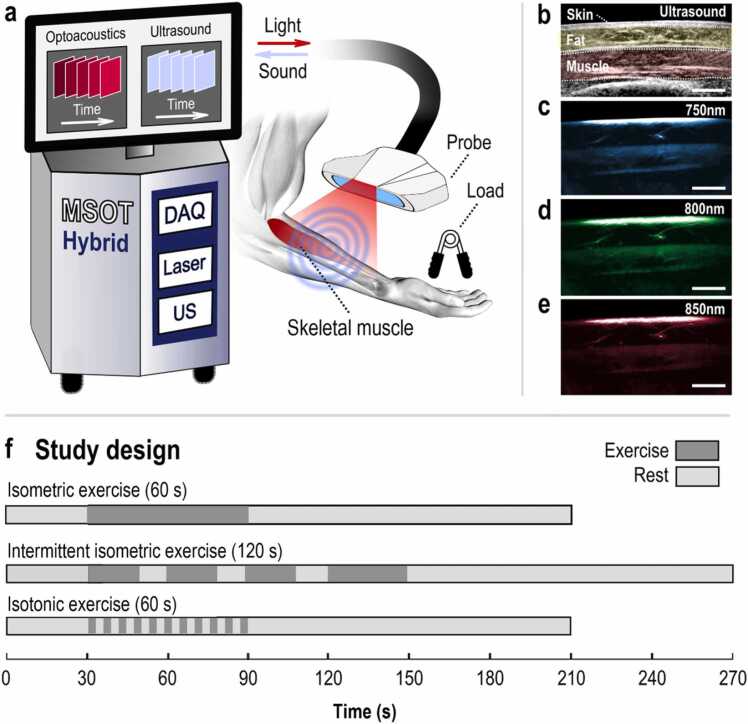
MSOT principle of operation and study design. (a) Hand-held hybrid MSOT/US system scanning the skeletal muscle of the forearm (wavelength range: 700–970 nm). During the scan, the MSOT probe stays in direct contact to the skin. For visualization purposes only, the MSOT probe is here depicted to be at a distance from the forearm. (b) Acquired tomographic ultrasound image showing the skin interface, the subcutaneous fat layer (yellow), and the brachioradialis muscle (red) in the forearm. (c-e) MSOT image of the same region corresponding to the distribution of (c) deoxygenated hemoglobin (Hb) (750 nm), (d) total hemoglobin (total blood volume, TBV) (800 nm), and (e) oxygenated hemoglobin (HbO_2_) (850 nm). Scale bars: 1 cm. (f) Study design. Four (n = 4) healthy volunteers performed isometric, intermittent isometric, and isotonic exercise in their forearm during MSOT/US imaging. (For interpretation of the references to color in this figure legend, the reader is referred to the web version of this article.)

A hybrid MSOT system with embedded ultrasound (US) imaging (Acuity©, iThera Medical GmbH, Munich, Germany) was employed for all measurements (Fig. 1a). The system is equipped with a customized hand-held probe for both tissue illumination and US sensing described in detail elsewhere [32]. We applied a periodic pattern of 28 different wavelengths (from 700 to 970 nm at steps of 10 nm) to illuminate tissue with short pulses of < 10 ns in duration at a rate of 25 Hz. For each single-wavelength light pulse, an optoacoustic frame was recorded (i.e., Single-Pulse-Per-Frame, SPPF operation mode). In parallel, co-registered ultrasound images were acquired at a rate of 8 Hz. This configuration yielded a MSOT: US frame ratio of 3:1. As shown in Fig. 1, the MSOT recordings lasted 210 s for the isometric and isotonic and 270 s for the intermittent isometric exercise test.

### 2.3 Image processing and data representation

Recorded US signals for each light pulse were reconstructed into planar MSOT images using a model-based approach with a non-negativity constraint [33], [34]. The forearm muscle region was semi-automatically segmented for all images of each exercise recording. To this end, manual segmentation of the muscle image was performed only in the first reconstructed image under ultrasound guidance (Fig. 1b) and by consensus from two experts. Subsequently, a customized semi-automated algorithm based on active contours performed the muscle image segmentation for all images of each exercise recording [29].

**Fig. 2:**
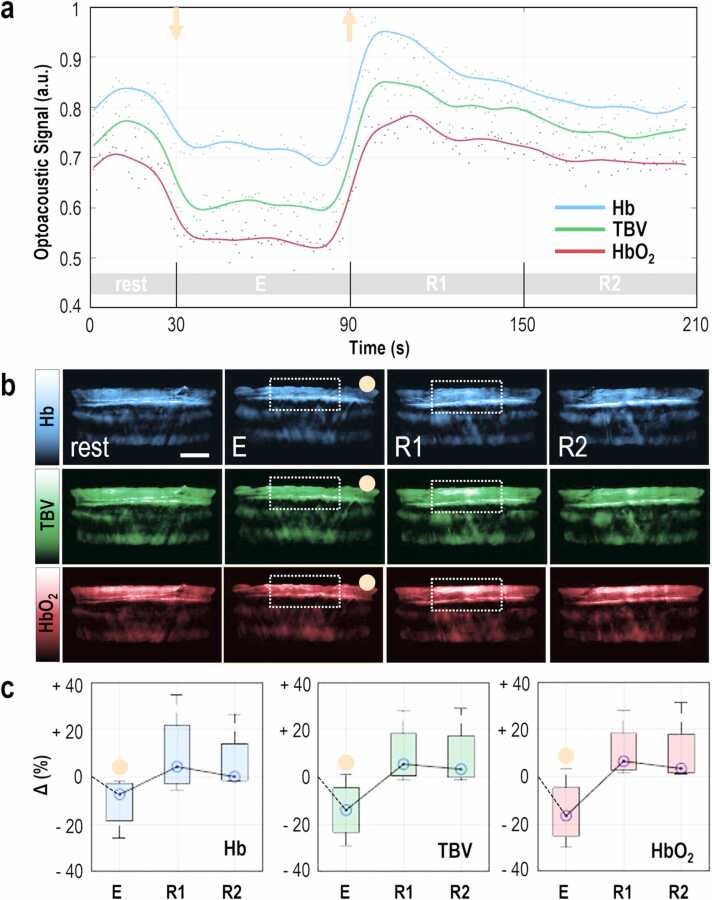
MSOT imaging before, during and after 60 s of isometric exercise. (a) Representative plot of the mean optoacoustic signal within the segmented brachioradialis muscle area, in the 750 nm MSOT frames for Hb, the 800 nm MSOT frames for TBV and the 850 nm MSOT frames for HbO_2_. TBV: total blood volume, rest: 30-second baseline period before isometric exercise, E: 60-second period of isometric exercise, R1: first 60-second resting period after exercise, R2: second 60-second resting period after exercise. The orange arrows show the start (down) and the end (up) of the contraction. (b) Top row: Representative 750 nm MSOT frames depicting the Hb-distribution in the segmented brachioradialis muscle for each time period described above. Middle row: Representative 800 nm MSOT frames depicting the TBV-distribution in the segmented brachioradialis muscle for each time period described above. Bottom row: Representative 850 nm MSOT frames depicting the HbO_2_-distribution in the segmented brachioradialis muscle for each time period described above. All frames show only the segmented muscle region. The white-dashed-line boxes show the regions of maximum signal change before and after the release of contraction. Scale bars: 1 cm. (c) Box plot of the mean change in Hb- (left), TBV- (middle) and HbO_2_- (right) optoacoustic signal within the muscle area for each period, with regard to the corresponding baseline value for all four (n = 4) subjects. The orange disks indicate the boxplots corresponding to a muscle contraction period. (For interpretation of the references to color in this figure legend, the reader is referred to the web version of this article.)

**Fig. 3:**
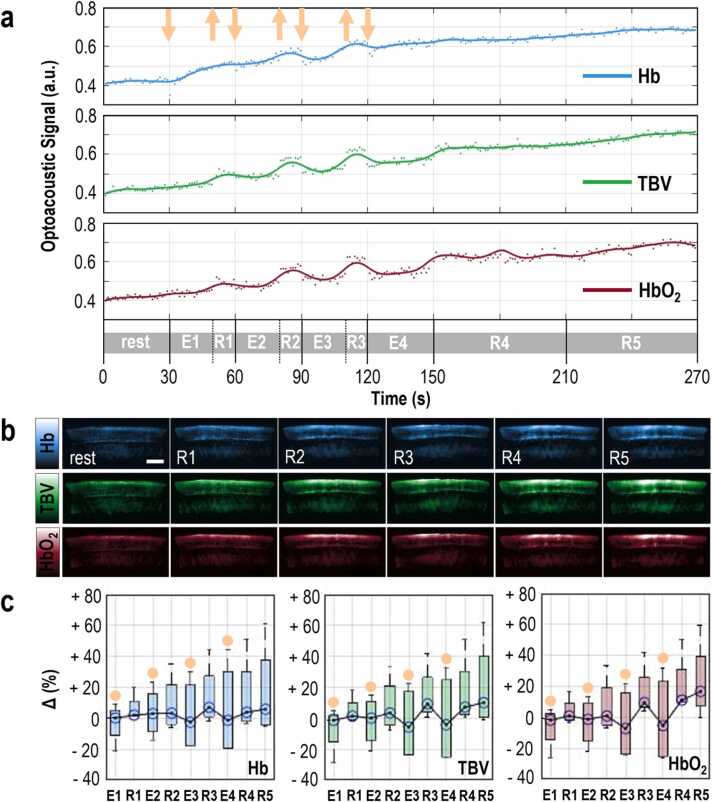
MSOT imaging before, during and after 120 s of intermittent isometric exercise. (a) Representative plot of the mean optoacoustic signal within the segmented brachioradialis muscle area, in the 750 nm MSOT frames for Hb, the 800 nm MSOT frames for TBV and the 850 nm MSOT frames for HbO_2_. TBV: total blood volume, rest: 30-second baseline period before isometric exercise, E1-E2-E3: 20-second period of isometric exercise, R1-R2-R3: 10-second resting period after each exercise period, E4: final 30-second exercise period, R4: first 60-second resting period after exercise, R5: second 60-second resting period after exercise. The orange arrows show the starts (down) and the ends (up) of the contractions. (b) Top row: Representative 750 nm MSOT frames depicting the Hb-distribution in the segmented brachioradialis muscle for each resting time period described above. Middle row: Representative 800 nm MSOT frames depicting the TBV-distribution in the segmented brachioradialis muscle for each resting period described above. Bottom row: Representative 850 nm MSOT frames depicting the HbO_2_-distribution in the segmented brachioradialis muscle for each time period described above. All frames show only the segmented muscle region. Scale bars: 1 cm. (c) Box plot of the mean change in Hb- (left), TBV- (middle) and HbO_2_- (right) optoacoustic signal within the brachioradialis muscle for each time period defined above, with regard to the corresponding baseline value for all four (n = 4) participating subjects. The orange disks indicate the boxplots corresponding to a muscle contraction period. (For interpretation of the references to color in this figure legend, the reader is referred to the web version of this article.)

To produce the subject-specific plots (e.g., Fig. 2a, Fig. 3a, etc.), we first calculated the mean intensity values within the segmented muscle areas for all MSOT images. Then, for each exercise recording, the calculated values in the 750 nm images were considered as the intramuscular Hb values (Fig. 1c), the values of the 800 nm images were considered as the intramuscular total blood volume (TBV) values (Fig. 1d), and the ones corresponding to the 850 nm images were taken as the intramuscular HbO_2_ values (Fig. 1e). The reason for this selection is that at 750 nm, the light absorption of Hb is prominently higher than that of HbO_2_; at 800 nm, the light absorptions of Hb and HbO_2_ are equal (isosbestic point); and at 850 nm, the light absorption of HbO_2_ is substantially higher than that of Hb [20]. All calculations took place in the ‘raw’ reconstructed optoacoustic frames, i.e. the recorded MSOT frames, before processing them for visualization purposes.

The images presented were processed further (denoised and contrast-enhanced to the same extent) for visualization purposes. To depict the maximum effect of each exercising (E) or resting (R) time period on the intramuscular hemodynamic parameters (Hb, TBV, HbO_2_), we selected the last recorded MSOT-frame of the corresponding period. The statistical boxplots of the whole group indicate the median, with the top edges of the box corresponding to the 25th and 75th percentiles, respectively.

### 2.4 Exercise protocol

To interrogate the capability of MSOT to assess muscle function and metabolism during exercise, we conducted three experiments with different types of exercise at intervals of 30 min (Fig. 1f). The first experiment examined muscle physiological responses to sustained isometric contraction with a stable workload (40 % of MVC). It was done by imaging the forearm for 30 s at resting state, 60 s during isometric hand-grip contraction, and 120 s of relaxation. The second experiment examined the response to intermittent isometric contraction and the physiologic effect of short relaxation periods between isometric contractions. The muscle was imaged for 30 s at resting state, 3 cycles of 20 s isometric contraction followed by 10 s relaxation, 30 s isometric contraction and finally 120 s of relaxation. Finally, the third experiment examined muscular functional and metabolic responses to isotonic exercise. The dorsolateral forearm was imaged 30 s before, 60 s during isotonic exercise at a rate of 1 Hz (1 contraction/s), and 120 s after the end of exercise.

## 3 Results

To investigate the patterns of circulatory function and oxygen metabolism within skeletal muscle during exercise, we conducted MSOT measurements in the brachioradialis muscle area during sustained handgrip tests. Three common types of exercise of gradual complexity were studied: i) isometric exercise: prolonged muscle contraction with stable intensity; ii) intermittent isometric exercise: short-duration muscle contractions with stable intensity interrupted by short-duration (e.g., 10 s) muscle relaxations; and iii) isotonic exercise: multiple fast muscle contractions (e.g., at ≈ 1 contraction/s) for a prolonged period (e.g., 60 s) with stable intensity.

Fig. 2 summarizes the results during isometric exercise which simulates the ‘quantum’ of all skeletal movements. The term ‘quantum’ is used here to describe the role of one single muscle contraction as ‘the minimum amount’ of the resulting muscle activity/effect, which produces a complex movement, e.g., of a limb. Fig. 2a shows a typical time course of HbO_2_, Hb and TBV (subject 1) at rest, during a 60 s sustained isometric handgrip exercise period (E) at 40 % maximum voluntary contraction (MVC) and throughout two (R1 and R2) 60 s resting periods. The plotted solid lines are fits of the measured MSOT values and represent the general trends for each measured hemoglobin parameter over time. An abrupt drop in TBV (−19.1 %) and HbO_2_ (−23.7 %) signals were observed at the onset of brachioradialis muscle contraction (Fig. 2a), while Hb (−15 %) decreased to a lesser extent (Fig. 2a). The contraction-induced reduction in Hb levels can be seen clearly in the respective MSOT images (Fig. 2b, top row). The drops in intramuscular TBV and HbO_2_ are also evident in the corresponding MSOT images (Fig. 2b, middle and bottom row) and are more prominent compared to the Hb images. After the initial drop, all hemoglobin parameters remained relatively stable for the entire 60 s period of isometric contraction (Fig. 2a). Upon muscle relaxation (t = 90 s), a rapid increase of all parameters was seen (≈ +3.8%/s for Hb, ≈ +3.6%/s for TBV, and ≈ +4.2%/s for HbO_2_). This phenomenon is also apparent in the corresponding MSOT Hb-, TBV-, and HbO_2_- images (Fig. 2b) showing a clear increase in signal amplitude over the muscle area. The recorded post-relaxation increase can be characterized as ‘reactive hyperemia’ since the hemoglobin maxima were clearly higher than the resting state. The reactive hyperemia lasted for ≈ 60–70 s and included a slow recovery period (≈ -0.4%/s for Hb, ≈ -0.4%/s for TBV and ≈ 0.2%/s for HbO_2_, between R1 and R2) for all hemoglobin variables. Fig. 2c illustrates the statistical changes of Hb, HbO_2_, and TBV over the exercise test for all subjects, as shown by the boxplot of the median and the 25th and 75th percentiles. Upon transition from rest to work (exercise), the Hb group median dropped by 7.3 % (Fig. 2c, left), the TBV median by 13.9 % (Fig. 2c, middle), and the HbO_2_ median by 16.5 % (Fig. 2c, right). Upon muscle relaxation (R1), a clear increase in the TBV group median, HbO_2_ and, to a smaller extent, Hb was observed. Table 1 provides a detailed overview of the fluctuations of the intramuscular MSOT-extracted hemodynamic parameters per exercise or resting period for all types of exercise for the whole group. All values express the percentage change of each period compared to the corresponding baseline value.

**Table 1:** Fluctuations of MSOT-extracted muscle parameters compared to the baseline for all volunteers. NHb (normalized Hb): Hb/TBV, NHbO_2_ (normalized HbO_2_): HbO_2_/TBV.

**Isometric Exercise**						
**Phase**	**Time (s)**	**Hb**	**NHb**	**TBV**	**HbO_2_**	**NHbO**_**2**_
E	60	-7.3%	+7.2%	-13.9%	-16.5%	-2.8%
R1	60	+4.3%	+0.9%	+5.4%	+6.5%	+0.4%
R2	60	+0.1%	-2.3%	+3.3%	+3.4%	+1.3%

**Intermittent Isometric Exercise**						
**Phase**	**Time (**s**)**	**Hb**	**NHb**	**TBV**	**HbO_2_**	**NHbO**_**2**_
E1	20	+0.2%	+3.1%	-1.4%	-1.8%	+1.0%
R1	10	+2.0%	+3.2%	+1.4%	+0.9%	-1.2%
E2	20	+2.9%	+4.9%	+0.1%	-1.3%	-1.3%
R2	10	+3.2%	+4.1%	+3.4%	+0.9%	-1.2%
E3	20	-2.5%	+3.5%	-5.7%	-7.3%	-1.3%
R3	10	+7.1%	+4.4%	+9.3%	+9.5%	-1.0%
E4	20	-1.3%	+4.1%	-4.2%	-5.4%	-1.0%
R4	60	+3.8%	-2.2%	+7.2%	+11.1%	+1.1%
R5	60	+5.8%	-3.4%	+10.1%	+16.7%	+0.8%

**Isotonic Exercise**						
**Phase**	**Time (**s**)**	**Hb**	**NHb**	**TBV**	**HbO**_**2**_	**NHbO**_**2**_
E1	20	+3.6%	+4.4%	+1.4%	-1.9%	-2.7%
E2	20	+15.9%	+7.7%	+10.9%	+8.5%	-4.5%
E3	20	+28.9%	+6.5%	+20.9%	+17.9%	-0.1%
R1	60	+22.1%	+0.1%	+22.4%	+21.8%	-0.8%
R2	60	+18.5%	-2.8%	+19.9%	+21.5%	+0.1%

In general, all MSOT-extracted hemodynamic parameters displayed similar kinetics over the testing period: a steep decline in signal amplitude at the onset of exercise, a relative steady state during the contractile phase, an exponential ‘hyperemic’ increase immediately after the release of contraction, and a gradual decrease during the recovery periods to near-baseline/resting levels.

As a next step, we investigated muscle function and metabolism during a more complex exercise pattern, intermittent isometric exercise, which includes periods of isometric contractions disrupted by periods of rest. Such an exercise pattern provides the opportunity to explore possible accumulative effects of repeated isometric contractions on muscle hemodynamics and oxygenation parameters. Fig. 3 summarizes the results for the intermittent isometric exercise challenge that consisted of four sequential forearm contraction periods (E1-E3 = 20 s and E4 = 30 s), three intervening rest periods (R1-R3 = 10 s) and two closing rest periods (R4-R5 = 60 s). Fig. 3a shows the MSOT-extracted Hb, TBV, and HbO_2_ changes as reported by the mean intensity pixels within the muscle area of a study participant (subject 3) throughout the testing period. For all extracted parameters, we observed a ‘cyclic’ pattern of decreases and increases corresponding to the contraction/blood washout and relaxation/hyperemia periods, respectively. Due to the four contraction/relaxation or blood washout/hyperemia cycles, the muscle seemed to gradually gain HbO_2_, TBV, and Hb in the course of the whole exercise challenge. All MSOT-extracted parameters increased in total, with their final levels (corresponding to the R5 resting period) clearly higher compared to baseline (e.g., for subject 3: ≈ +48.9 % for Hb, ≈ +39.1 % for TBV and ≈ +43.4 % for HbO_2_). During the intermittent isometric exercise of subject 3, the intramuscular Hb, TBV, and HbO_2_ increased at a rate of ≈ +0.4%/s, ≈ +0.3%/s and ≈ 0.4%/s respectively, yet at a much slower rate compared to the rate following the end of the isometric exercise. The recorded outcome represents the accumulative effect of consecutive isometric contraction periods which are characterized by a transient decrease of all parameters, as described above. Our results are supported by the corresponding single-wavelength MSOT images for Hb (750 nm), TBV (800 nm), and HbO_2_ (850 nm) (Fig. 3b). An overview of the dynamics of the MSOT-extracted parameters during the intermittent isometric exercise challenge for the whole cohort is provided in Table 1 (Fig. 3c).

**Fig. 4:**
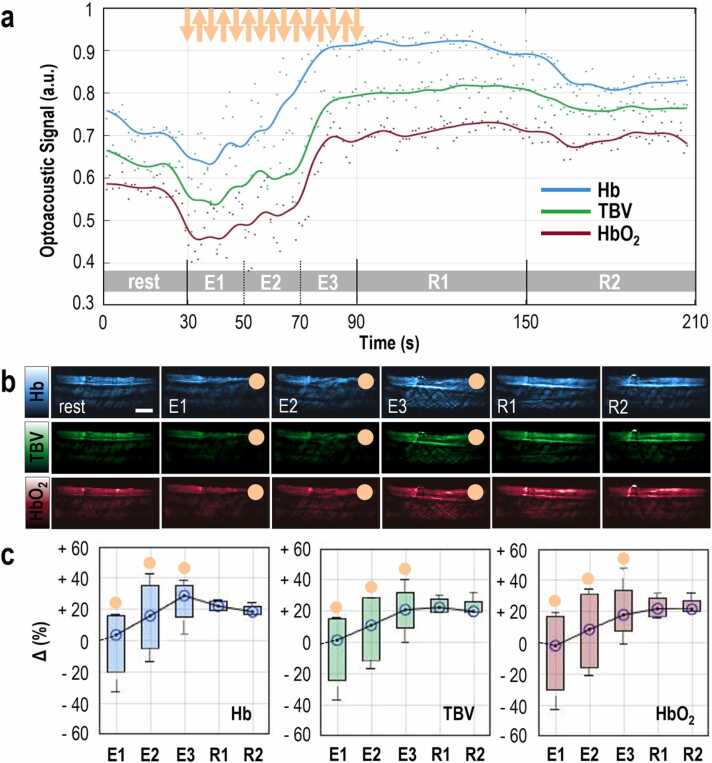
MSOT imaging before, during and after 60 s of isotonic exercise. (a) Representative plot of the mean optoacoustic signal within the segmented brachioradialis muscle area, in the 750 nm MSOT frames for Hb, the 800 nm MSOT frames for TBV and the 850 nm MSOT frames for HbO_2_. TBV: total blood volume, rest: 30-second baseline period before isometric exercise, E1-E2-E3: 20-second subperiods of the total 60-second period of isotonic exercise, R1: first 60-second resting period after exercise, R2: second 60-second resting period after exercise. The orange arrows show roughly the starts (down) and the ends (up) of the contractions. (b) Top row: Representative 750 nm MSOT frames depicting the Hb-distribution in the segmented brachioradialis muscle for each time period described above. Middle row: Representative 800 nm MSOT frames depicting the TBV-distribution in the segmented brachioradialis muscle for each time period described above. Bottom row: Representative 850 nm MSOT frames depicting the HbO_2_-distribution in the segmented brachioradialis muscle for each time period described above. All frames show only the segmented muscle region. Scale bars: 1 cm. (c) Box plot of the mean change in Hb- (left), TBV- (middle) and HbO_2_- (right) optoacoustic signal within the brachioradialis muscle for each period, with regard to the corresponding baseline value for all four (n = 4) participating subjects. The orange disks indicate the boxplots corresponding to a muscle contraction period. (For interpretation of the references to color in this figure legend, the reader is referred to the web version of this article.)

As a final experiment, we performed MSOT measurements during isotonic contractions (Fig. 4), which are the type of contractions in aerobic (e.g., running, swimming) and resistance training (e.g., pushups, squats) exercise. These kinds of exercise are the most common in both health-related exercise and rehabilitation schemes. Readouts of subject 1 show a very short period (≈ 8 s, Fig. 4a) of decrease of all intramuscular parameters, followed by a relatively monotonic and fast increase of Hb, TBV, and HbO_2_ (≈ +0.9%/s for Hb, ≈ +1%/s for TBV and ≈ +1.1%/s for HbO_2_) during the exercise phase, a phenomenon reflecting the cumulative effect of periodic muscle contraction and relaxation at a rate of ≈ 1 Hz. Furthermore, during the first minute after the end of exercise, all parameters remained roughly stable, indicating a clearly longer hyperemic plateau with a much longer hyperemic period (> 120 s) compared to the isotonic exercise readout of the same subject (≈ 60–70 s). Corresponding MSOT image sequences (Hb, TBV, and HbO_2_) for the baseline resting phase, three 20 s exercise (E1-E3) and two 60 s (R1, R2) post-exercise resting periods are given in Fig. 4b. A detailed record of the statistics of the hemodynamic fluctuations (compared to baseline) presented in Fig. 4c is provided in Table 1.

**Fig. 5:**
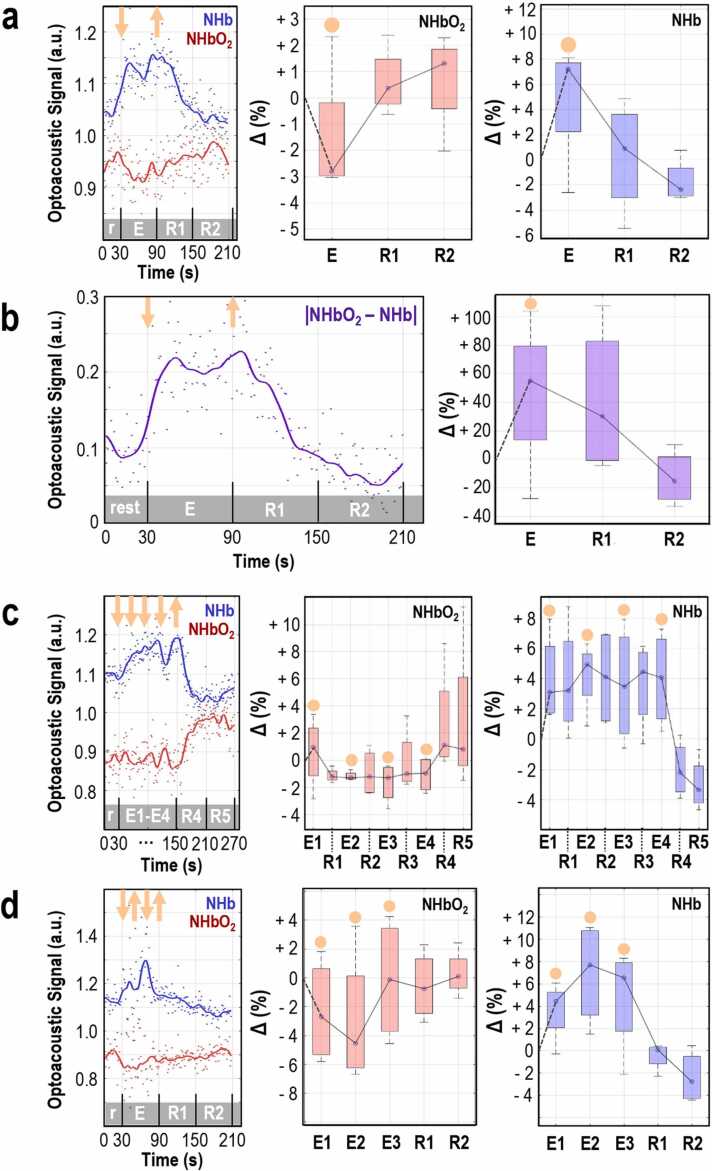
MSOT-estimation of muscle oxygen kinetics during exercise. (a) Left: Exemplary plot of normalized HbO_2_ (NHbO_2_ = HbO_2_/TBV) and normalized Hb (NHb = Hb/TBV) in muscle during 60 s of isometric contraction, box plot of the mean change in NHbO_2_ (middle) and NHb (right) in the measured muscle for each time period defined above, with regard to the corresponding baseline value for all four participating subjects. The orange arrows show the start (down) and the end (up) of the contraction. The orange disks indicate the boxplots corresponding to a muscle contraction period. (b) Left: Representative plot of the absolute difference between the normalized HbO_2_ (NHbO_2_ = HbO_2_/TBV) and the normalized Hb (NHb = Hb/TBV) within the muscle, right: Box plot of the mean change in MSOT-estimated AV oxygen difference within the forearm muscle for each period, with regard to the corresponding baseline value for all four participating subjects. (c) Left: Exemplary plot of NHbO_2_ and NHb in muscle during intermittent isometric exercise (3 sets of 20-second contraction and 10-second rest and 1 set of 30-second contraction), box plot of the mean change in NHbO_2_ (middle) and NHb (right) in the measured muscle for each time period defined above, with regard to the corresponding baseline value for all subjects. The orange arrows are roughly indicative of the exercise pattern due to space limitations. (d) Left: Exemplary plot of NHbO_2_ and NHb in muscle during 60 s of isotonic exercise, box plot of the mean change in NHbO_2_ (middle) and NHb (right) in the measured muscle for each time period defined above, with regard to the corresponding baseline value for all (n = 4) subjects. The orange arrows are roughly indicative of the exercise pattern due to space limitations. (E: exercise, R: rest). (For interpretation of the references to color in this figure legend, the reader is referred to the web version of this article.)

To further investigate the intramuscular patterns of arterial and venous circulation in exercise and their association with local oxygen kinetics, we conducted an additional analysis of the MSOT readouts. Fig. 5 depicts the dynamics of Hb and HbO_2_ after their normalization to TBV, for all types of exercise_._ These normalized parameters (NHb and NHbO_2_) are indicators of the relative changes between the deoxygenated (Hb) and total (TBV) hemoglobin or the oxygenated (HbO_2_) and total hemoglobin, respectively. In particular, by monitoring the NHb and NHbO_2_ we could further highlight the exercise-induced circulatory function in the muscle or the relative changes in muscle venous and arterial blood, considered as fractions of the TBV. Compared to the fluctuations of the non-normalized Hb and HbO_2_ presented in Fig. 2, Fig. 3, Fig. 4, the NHb (Hb/TBV) and NHbO_2_ (HbO_2_/TBV) follow different patterns. For example, during isometric exercise (Fig. 5a), NHb is characterized by an increase while NHbO_2_ by a decrease. A complete record of the percentage change for each normalized parameter with reference to the corresponding baseline is provided in Table 1. The effect of diverging curves or behaviors observed in Fig. 5a is further highlighted in Fig. 5b, where the absolute difference between NHbO_2_ and NHb (|NHbO_2_-NHb|) is depicted. |NHbO_2_-NHb| is an indicator of the difference between the venous and the arterial fractions of muscle TBV or the arterio-venous difference in hemoglobin oxygenation / oxygen content. Such *a* parameter may reflect the muscle oxygen utilization during, or in response to, contraction. As observed, |NHbO_2_-NHb| for subject 3 (Fig. 5b) increased during isometric contraction and decreased after muscle relaxation, reaching the resting levels within the first minute of rest. Fig. 5b (right) provides the corresponding statistics for all subjects: |NHbO_2_-NHb| showed an increase of +55.2% compared to its baseline level during exercise (E), remained increased (+30.1 %) during the first post-exercise resting minute (R1) and clearly decreased (−15.2 %) compared to baseline two minutes after the start of muscle relaxation (R2). The corresponding normalized data for the intermittent isometric and isotonic exercises are presented in Fig. 5c and d and are also detailed in Table 1.

## 4 Discussion

Muscle contractions/exercise are regulated by interrelated mechanical (e.g., increase of intramuscular pressure and stresses) and metabolic (e.g., oxygen exchange and nutrient uptake) events. In this work, we demonstrate that label-free muscle optoacoustic imaging can provide a direct view into muscle function and metabolism. In particular, we showcase the capability of hand-held MSOT to directly image skeletal muscle hemodynamics and oxygenation during different types of exercise by spatially and temporally monitoring Hb and HbO_2_ levels and offering detailed maps of muscle functional and metabolic parameters. These capabilities allow for the quantification of muscle performance and optimization in a training scheme. In addition, it opens possibilities for the direct examination of the target organ in a wide range of diseases which may affect the peripheral vasculature (e.g., PAD), general metabolism (diabetes mellitus), cardiovascular system (e.g., heart failure), or musculature (e.g., muscular dystrophies, metabolic myopathies).

Muscle blood perfusion is the most important parameter influencing muscle function and is directly associated with TBV changes [35]. Muscle perfusion consists of arterial and venous components and is thus directly related to intramuscular HbO_2_ and Hb levels. MSOT can be used to detect TBV, HbO_2_, and Hb levels, and thus monitor all main parameters of intramuscular circulation in a single test. In our study, the onset of forearm muscle contraction clearly provoked an abrupt drop of Hb, HbO_2_, and TBV in all three experimental protocols tested, although the intensity of the effect was influenced by the duration of the contraction. Accordingly, the rapid decline in all parameters in response to contraction was most pronounced in the first experimental setting (Fig. 2) which involved a single continuous 60 s isometric contraction. This hemodynamic response is typical of sustained isometric muscle contractions [36] and is thought to result from increases in intramuscular pressure [37] during contraction, causing obstruction of arterial blood inflow and increased venous blood outflow induced by the muscle pump (also known as “blood spurts”) [38]. Indeed, it has been previously reported that isometric MVC provokes a complete obstruction of muscle blood flow/perfusion in the vastus lateralis muscle [39]. In our experiments with the single 60 s MVC, the continuous obstruction in arterial and venous circulation is also demonstrated by the fact that the localized muscle Hb, HbO_2_, and TBV levels remained stable during the entire duration of the contraction after an immediate initial drop. During recovery, the muscle demonstrated a rapid hyperemia phase, due to post-contraction vasodilation [40], followed by a slower recovery to near baseline levels.

Our results reflect the function of the blood circulation during muscle contraction: a series of hydraulic/mechanical phenomena driven by the strong intramuscular pressure due to contraction. During MVC, the pressure within the muscle may increase by up to 6000 % compared to the resting state (< 15 mmHg), reaching values of up to 250–1000 mmHg depending on the muscle size [41], [42]. Thus, pressures of even 40 % of the MVC (100–400 mmHg) are around or above the systolic blood pressure of a healthy or hypertonic subject. Therefore, both the arterial and venous flow significantly decrease under the effect of the pressure applied on the vascular wall during contraction. This effect leads to i) a significant decrease in arterial blood inflow, ii) a significant decrease in venous blood outflow, and iii) washout of the blood filling the vascular bed of the muscle just before the contraction. These previously described physiologic phenomena have been well captured and corroborated by the MSOT-extracted parameters.

Muscles not only perform sustained contractions, but also repetitive contractions interspersed with brief resting periods, which we observed during intermittent isometric (Fig. 3) and isotonic exercise (Fig. 4). However, TBV, Hb, and HbO_2_ tended to progressively increase with each contraction-relaxation cycle and even throughout recovery, suggesting a more sustained/frequently triggered hyperemic response compared with the single contraction experiment (Fig. 1). A likely explanation is that the mechanical hindrance to blood flow that occurred during contraction was too low to cause complete suppression of arterial blood inflow, which in combination with the post-contraction hyperemia, resulted in accumulation of blood in the muscle vascular bed with each successive exercise.

The effect of the different exercise schemes/types on the muscle hemodynamic response is also explored and visually described in the provided figures. In Fig. 2 (isometric exercise) the muscle has the time to completely relax so that all hemodynamic and oxygenation parameters come back to the previous situation. On the contrary, in Fig. 3 (intermittent isometric exercise), the time given to the muscle to relax between the exercise periods (20 s each) is short (10 s each). Thus, at the start of each relaxing period the muscle is slightly more perfused/hyperemic compared to the ‘global’ resting baseline, so that finally (after all exercise cycles) the muscle is much more hyperemic compared to the end of a simple isometric exercise period, for example, and the hyperemia phase much more prolonged. If we had continued the recording, the values would have started reaching the resting state soon. This ‘incremental’ increase of the hemodynamic and oxygenation parameters is also observed in the Fig. 4 (isotonic exercise), albeit at a faster rate, where the muscle gains blood between each contraction (the muscle contracts at rates of almost 1 Hz) and, hence, needs more time to return to the baseline values, compared to the simple isometric exercise of Fig. 2.

Because exercise induces vasodilation and increases blood perfusion to meet the increased metabolic/oxygen demand, muscle contraction extends beyond a simple mechanic/hydraulic event. Of great importance are: i) the relative changes of HbO_2_ and Hb levels and ii) the absolute arteriovenous oxygen difference, which are both strong indicators of oxygen exchange kinetics between the muscle and the vessels or the muscle oxygen uptake during exercise.

The relative changes in Hb and HbO_2_, as expressed by the normalized parameters NHb and NHbO_2_, reveal a different aspect of muscle function during exercise. These data indicate relative changes in intramuscular Hb and HbO_2_ levels independent of TBV changes and give insights into the course of arteriovenous oxygen difference and, thus, oxygen exchange/kinetics over the course of sustained contractions. We observed that during sustained isometric contraction, NHb increased while NHbO_2_ decreased, reflecting a slight deoxygenation trend during contraction within the measured muscle. We also explored the absolute difference between NHbO_2_ and NHb and observed that this difference increased dramatically and then plateaued throughout the sustained contraction. Our observation may well reflect a metric of the muscle extracting oxygen from the blood. Following muscle relaxation, the difference in Hb and HbO_2_ signals rapidly returned to baseline resting levels. Likewise, the cyclical pattern in oxygenation-deoxygenation observed during the respective contraction-relaxation periods suggests that increased oxygen extraction was primarily related to the contractile phase of the cycle.

In the current study, we explored the circulatory phenomena and oxygen metabolism taking place during exercise of the brachioradialis muscle: a fast twitch muscle. Based on previous experience using MSOT to image hemoglobin gradients under disturbed blood flow within the gastrocnemius muscle, which contains 50 % slow twitch fibers [43], future MSOT studies could provide further insights into the possible differences in oxygen metabolism between fast and slow twitch muscles [44].

It is important to acknowledge some of the limitations of our study, such as the small sample size and the lack of data validation using independent methods that assess muscle activity. Future MSOT studies combined with MRI/MRS, CEUS, electromyography, NIRS, and other relevant techniques could provide valuable insights into muscle energetics and further assess the sensitivity of MSOT in capturing hemodynamic and oxygenation changes in muscles during exercise or disease. Furthermore, MSOT measurements might be characterized by variations, as for example seen in Fig. 2, Fig. 4, during the baseline as well as exercise and recovery phases. We believe that this could be the effect of possible laser light energy variations. It is indeed true that the laser output energy may slightly vary among different wavelengths/pulses or over time. Despite, the slight variations, our data show a generally stable behavior of the laser even for long recordings. However, the manufacturer has taken into account of this effect and a compensation mechanism has been implemented. Thus, the recorded images are weighted with the laser energy output so that the effect of different illumination energy on the image intensities is considered to be negligible.

## 5 Conclusion

This is the first investigation using MSOT to monitor and quantify changes in hemodynamic and oxygen levels in skeletal muscle during isometric/isotonic resistance training. Our results demonstrate that MSOT can provide both sensitive and muscle-specific perfusion and oxygenation values, offering unique insights into muscle function and metabolism by direct imaging of ‘aerobic’ processes. Our data are in good agreement with prior investigations of muscle hemodynamics and oxygenation responses to resistance training while offering several benefits over other techniques, suggesting that MSOT could become an invaluable tool in the evaluation of exercise performance. By offering non-invasive, label-free, continuous monitoring of tissue perfusion and oxygenation of exercising muscle, MSOT could increase our understanding of the complex training variables on performance and aid in establishing training recommendations in health and disease. We anticipate that MSOT imaging of muscle activity will play an important role in sports science by improving the monitoring of athletic performance, rehabilitation and overall healthcare of patients with relevant diseases.

## Funding

This project received funding from the 10.13039/501100000781European Research Council (ERC) under the European Union’s Horizon 2020 research and innovation programme under grant agreement No 694968 (PREMSOT), the DZHK (10.13039/100010447German Centre for Cardiovascular Research; FKZ 81Z0600104) and the 10.13039/501100013295Helmholtz Zentrum München funding program “Physician Scientists for Groundbreaking Projects”.

## CRediT authorship contribution statement

**Angelos Karlas:** Conceptualization, Methodology, Formal analysis, Investigation, Visualization, Funding acquisition, Data curation, Supervision, Writing - original draft, Writing - review & editing. **Nikolina-Alexia Fasoula:** Methodology, Formal analysis, Investigation, Data curation, Writing - original draft, Writing - review & editing. **Nikoletta Katsouli:** Methodology, Data curation, Formal analysis, Writing - original draft, Writing - review & editing. **Michael Kallmayer:** Investigation, Methodology, Writing - review & editing. **Sabine Sieber:** Writing - review & editing. **Sebastian Schmidt:** Writing - review & editing. **Evangelos Liapis:** Formal analysis, Validation, Writing - original draft, Writing - review & editing. **Martin Halle:** Supervision, Writing - review & editing. **Hans-Henning Eckstein:** Supervision, Resources, Writing - review & editing. **Vasilis Ntziachristos:** Supervision, Funding acquisition, Conceptualization, Methodology, Resources, Validation, Writing - review & editing.

## Declaration of Competing Interest

The authors declare the following financial interests/personal relationships, which may be considered as potential competing interests: Vasilis Ntziachristos reports a relationship with sThesis GmbH that includes: equity or stocks. Vasilis Ntziachristos reports a relationship with iThera Medical GmbH that includes: equity or stocks. Vasilis Ntziachristos reports a relationship with Spear UG that includes: equity or stocks. Vasilis Ntziachristos reports a relationship with i3 Inc. that includes: equity or stocks. All other authors declare that they have no competing interests.

## Data Availability

Data will be made available on request.
